# Cholera

**Published:** 1854-09

**Authors:** 


*Cholera.*—The epidemic is on the decline, and with it the total number of deaths from all causes. During the week ending August 26th, there were but forty-three deaths from cholera, and one hundred and twenty-six from all causes.

We have at no time had much over 70 deaths per week, and that for only two weeks. The cooler weather, and fresher breezes which we may soon expect, will probably reduce the mortality to its average standard.

We cannot learn that anything new has been elicited in the treatment of cholera. Aside from the standard treatment by calomel and opium, the sulphuric acid treatment, and that by comparatively large doses of strychnia, were the most prominent candidates for favor at the early part of the season. The strychnia disappointed the expectations of its friends. In any safe dose its effect was not noticeable, while if too much was given, as happened in the hands of the Homœopathists, the patients added tetanus to their list of symptoms.

The sulphuric acid was also a failure in most hands. Whether from want of skill in its exhibition, or of adaptation to cases, most of our friends who used it are inclined to fall back upon first principles. Others claim to have derived much benefit from its use, not in true cholera, but in some of the allied forms of bowel disease. It has certainly failed to sustain the expectations excited by the English reports of its success. It is probably somewhat out of its latitude here.

The calomel question is still open. Its advocates have given less than in previous years, while its opponents have probably given rather more. A dose of over ten grains has been very uncommon, while it has been a very general practice to give that quantity, with a grain of morphia, as a first prescription. The calomel is rarely repeated, while the morphia is usually given in smaller doses until the vomiting and purging cease. Of course, the friends of the calomel ascribe the curative effect to it, while the advocates of the morphia think that it has quite as likely been the active agent in the prescription. How to settle the question no one can tell, for no one gives the calomel alone, while but a few omit it entirely. One thing is noteworthy, the patients do not readily come under the mercurial action. We have seen no one salivated, neither have we noticed the other peculiar effects upon the hepatic secretion. The course of convalescence was usually a cessation of the vomiting and purging, a return of the warmth of the skin, and a general improvement in appearance. It was remarkable that bilious or dark colored evacuations were not necessary to this result. Frequently the last discharge would be rice water, and the patient would go on convalescing for days without a movement of any kind.

Of the treatment by small and repeated doses we know but little. It has probably found few friends, as we have heard nothing of it. It belied its promises two years ago, and the profession had little confidence in it.

The mainstay of all methods of treatment has really been opium in moderately large doses, combined with diffusible stimulants, counter-irritation, and hot air. Perturbation by friction has been found injurious.

There has been an unusual tendency to congestive disturbance. Contrary to previous experience, it has been found that a cessation of discharges by no means implied recovery. In very many cases where the discharges had been too small to account for unusual prostration, the patient has sunk inevitably into a collapse too deep for remedial measures. There was such an entire want of nerve-force that no treatment was of any avail.

Congestion of the brain followed in a large number of cases. It was at first thought that this was attributable to too free use of opiates; but subsequent experience, based upon the withdrawal of them from the treatment, has proved that it was an essential feature of the disease, and not peculiar to any method of treatment.

The irregulars—the homœopathists, eclectics, etc.,—have been unusually quiet. Judging from the record of the Board of Health, they have had but little to do with the disease, and that little has not been attended by any degree of success. As in most severe epidemics, the regular physicians have done the labor and are entitled to any credit which may be derived from its success. If our illegitimate cousins commence their usual boastings at the close of the epidemic, it is only necessary to refer to the official records.

In one instance a homœopath having boasted of curing *thirty-four* cases of cholera, was immediately visited by a health-inspector, with a warrant requiring him to show cause why he had not reported them according to law. Of course there was an immediate falling off of homœopathic success; the "thirty-four" cases of cholera dwindling down to "some" cases of diarrhœa. This necessity of reporting cases has been a great stumbling block to quackery. Early in the season they began by neglecting to report their fatal cases. A few of them were fined by the Police Justice, and the result has been that they have cut a very small figure in the medical world.

It is fortunate that reliable statistics exist with which legitimate medicine may stop the boasting of pretenders. It will be interesting to compare results at the end of the season.

We expect to publish in a coming number a communication from a gentleman whose opportunities for careful observation have been very large, in which the sequelae of cholera will be considered at length, with reference to their treatment. H.

